# A Pilot Clinical Study of Hyperacute Serum Treatment in Osteoarthritic Knee Joint: Cytokine Changes and Clinical Effects

**DOI:** 10.3390/cimb43020046

**Published:** 2021-07-09

**Authors:** Isabel Olmos Calvo, Eszter Fodor, Dorottya Kardos, István Hornyák, Adél Hinsenkamp, Olga Kuten-Pella, Zsuzsanna Gyevnár, Gábor Erdélyi, Tamás Bárdos, Tamás Mirkó Paukovits, Krisztián Magos, György Béres, Stefan Nehrer, Zsombor Lacza

**Affiliations:** 1 OrthoSera GmbH, Dr. Karl-Dorrek-Straße 23-29, 3500 Krems an der Donau, Austria; olga.kuten@orthosera.com; 2 Department of Sport Physiology, University of Physical Education, 44 Alkotás utca, 1123 Budapest, Hungary; eszter.fodor@orthosera.com (E.F.); gyevnarzs@gmail.com (Z.G.); zsombor.lacza@orthosera.com (Z.L.); 3 Institute of Translational Medicine, Semmelweis University, 26 Üllöi utca, 1085 Budapest, Hungary; dorottya333@gmail.com (D.K.); istvan.hornyak@orthosera.com (I.H.); adel.hinsenkamp@orthosera.com (A.H.); 4 Kastélypark Klinika, 15 Hajdú utca, 2890 Tata, Hungary; ergab66@gmail.com (G.E.); tamasbardos74@gmail.com (T.B.); tamasmirkopaukovits@gmail.com (T.M.P.); drmagoskrisztian@gmail.com (K.M.); gyorbe@gmail.com (G.B.); 5 Center for Regenerative Medicine, Danube University, Dr. Karl-Dorrek-Straße 30, 3500 Krems an der Donau, Austria; stefan.nehrer@donau-uni.ac.at

**Keywords:** knee osteoarthritis, hyperacute serum, cytokines, VAS, KOOS, Lysholm-Tegner, matrix metalloprotease, interleukin

## Abstract

The serum fraction of platelet-rich fibrin (hyperacute serum) has been shown to improve cartilage cell proliferation in in vitro osteoarthritic knee joint models. We hypothesize that hyperacute serum may be a potential regenerative therapeutic for osteoarthritic knees. In this study, the cytokine milieu at the synovial fluid of osteoarthritic knee joints exposed to hyperacute serum intraarticular injections was investigated. Patients with knee osteoarthritis received three injections of autologous hyperacute serum; synovial fluid was harvested before each injection and clinical monitoring was followed-up for 6 months. Forty osteoarthritic-related cytokines, growth factors and structural proteins from synovial fluid were quantified and analysed by Multivariate Factor Analysis. Hyperacute serum provided symptomatic relief regarding pain and joint stability for OA patients. Both patients “with” and “without effusion knees” had improved VAS, KOOS and Lysholm-Tegner scores 6 months after of hyperacute serum treatment. Synovial fluid analysis revealed two main clusters of proteins reacting together as a group, showing strong and significant correlations with their fluctuation patterns after hyperacute serum treatment. In conclusion, hyperacute serum has a positive effect in alleviating symptoms of osteoarthritic knees. Moreover, identified protein clusters may allow the prediction of protein expression, reducing the number of investigated proteins in future studies.

## 1. Introduction

Osteoarthritis (OA) is a degenerative musculoskeletal disease that mainly affects weight-bearing joints leading to articular cartilage and subchondral bone destruction. Knees are one of the most affected joints in OA, reaching a prevalence of 3.64% of the worldwide population (which equals to 250 million people) [1]. With an expected increase in human lifespan, the prevalence of OA will rise in the next decades, leading to a significant economic burden increment. Nowadays, once the OA joint is injured, pharmacologic treatments are focused on alleviating symptoms and as a last resort, the joint is replaced. Intraarticular (IA) joint injections with different therapeutics such as hyaluronic acid (HA) or platelet-rich plasma (PRP) are used as a pre-operative therapy and have been widely studied [2,3].

PRP is a blood-derived product that has become the focus of attention as potential first-line therapy in OA [4,5]. Several studies have shown that PRP has beneficial effects on pain and mobility when injected into the knee joint at various stages of knee degeneration [6]. Similar results were published for other blood derivatives such as autologous conditioned serum, plasma rich in growth factors and more recently autologous protein solution [7,8,9,10,11,12]. The published body of evidence is reaching a point that some physicians now consider PRP their first choice for non-operative treatment [13]. However, some large and well-controlled studies failed to confirm the positive effects seen in smaller cohorts, raising doubts about whether it is justified to widen the use of PRP to the general OA population [14,15,16,17]. A key issue that hinders further protocol optimization is the unclear mode of action [18]. Therefore, in vitro models of the osteoarthritic joint are helpful to understand what exactly is to be expected from blood derivative treatments [19,20]. Moreover, there is controversy about the lack of standardization of PRP production, leading to multiple formulations and variability [21].

This has been overcome by the development of hyperacute serum, a similar blood-derived product created by centrifuging freshly drawn blood in a glass tube, which activates the coagulation cascade. A fibrin clot that traps exclusively aggregated platelets is formed. By pressing this fibrin clot, hyperacute serum is extracted, in a process that takes 8 min from blood drawing, before the inflammatory process begins. In our previous studies we have extensively investigated PRP varieties and observed that hyperacute serum has a more reliable effect than PRP when comparing proliferation of various cells and tissues [22,23]. This serum preparation differs from PRP by the harvesting and the activation route and has a different growth factor and cytokine composition [22,24,25]. Previous experimental studies showed that the supplementation of hyperacute serum to cell culture medium has proliferative effects on bone, cartilage and mesenchymal stem cells and can restore the regenerative potential of the bone marrow niche to a similar extent than bone morphogenic proteins [26]. The regenerative effect of hyperacute serum was observed not just at the cellular level but also in the complete osteoarthritic joint. *Kardos* et al., performed a study with hyperacute serum on an in vitro explant co-culture model of OA knee and showed promising results in human studies [19].

To assess firstly, the safety and feasibility; and secondly, the possible benefits from hyperacute serum IA treatment in OA knees, patient reported outcomes together with synovial fluid samples were gathered. Lysholm-Tegner score, visual analog scale (VAS) and the knee injury and osteoarthritis outcome score (KOOS) are commonly used indicators for similar clinical studies in the context of OA [27,28,29,30,31]. Synovial fluid is an easily harvestable specimen that has the potential of been used as an indicator in the stage of the disease. Previous studies investigated the correlation between cytokine levels in the synovial fluid and the severity of OA [32,33]. Multiple cytokines have been shown to play a role or to be up- or downregulated during OA; however, neither one of them has revealed a strong enough correlation to be used as a unique marker for the disease or for the prediction of a treatment. Therefore, a group of 40 proteins relevant in OA joints were analyzed in this study (Suppl. Table S1) [32,33,34,35,36,37,38,39,40,41,42,43,44,45,46,47,48,49,50,51,52,53,54,55,56,57,58,59,60,61,62,63].

In the current clinical study, hyperacute serum was firstly used in vivo OA patients showing that IA administration of the mentioned therapeutic was not only safe but also induced a clinical improvement of OA knees. Protein changes induced at the synovial fluid after hyperacute serum therapy were quantified and analyzed to better identify and understand its molecular mode of action.

## 2. Materials and Methods

### 2.1. Hyperacute Serum Isolation

**Figure 1:**
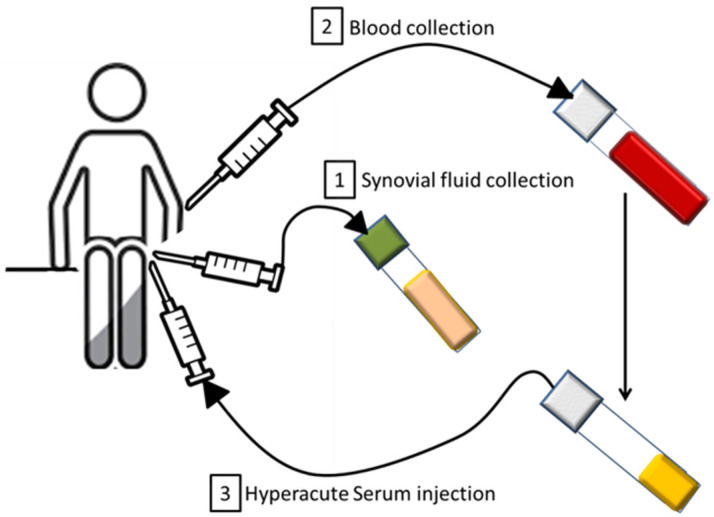
Schematic of the medical procedure. Firstly, when possible, synovial fluid was collected from the patient’s knee. Then, blood was taken and hyperacute serum was obtained from it. Hyperacute serum was finally intraarticularly injected.

Blood samples were obtained from patients of both sexes aged 24–58 years. Eighteen ml venous blood was taken by venipuncture from the patients with the hypACT Inject device (OrthoSera Kft, Budapest, Hungary), which was immediately centrifugated at 1710× *g* for 8 min [64]. After centrifugation, the red blood cell containing fraction was removed and hyperacute serum was pressed out from the clotted platelet rich fibrin clot and given back to the patient’s knee joint right after centrifugation (Figure 1).

### 2.2. Intraarticular Serum Treatment

**Figure 2:**
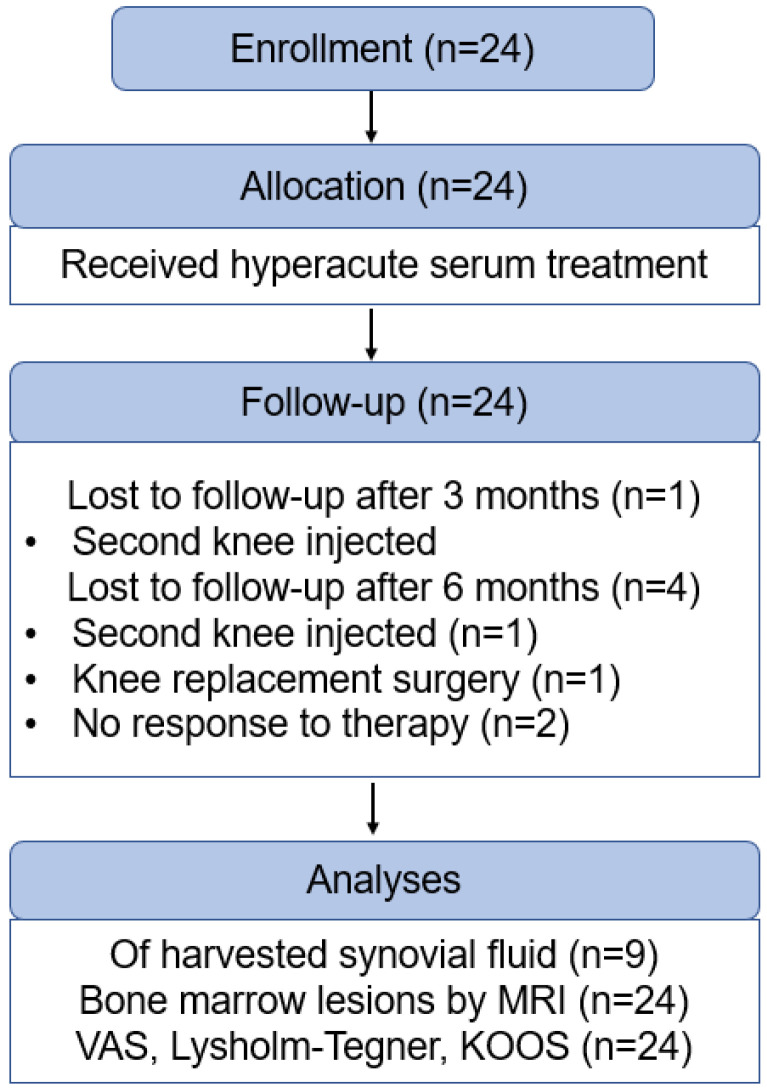
Flow diagram of participant enrolment, allocation, follow-up and analysis of the study trial.

Patients (*n* = 24) with knee osteoarthritis received three intraarticular injections of 3 mL autologous hyperacute serum at weekly intervals (IRB approval number 6909/2017/EKU; on the 17 February 2017). Inclusion and exclusion criteria are shown in Suppl. Table S2 and patient information in Suppl. Table S3. Before each injection, withdrawal of excess synovial fluid was attempted and when available it was immediately frozen and kept at −80 °C until cytokine analysis. Clinical signs of acute inflammation were registered by palpation and surface heat mapping. Pain (VAS) and mobility (Lysholm-Tegner and KOOS) were monitored at each office visit, e.g., before, after 1 and 2 weeks and at 3- and 6-months post-injection (Figure 2).

### 2.3. Cytokine Measurements

A literature review on cytokines, growth factors and other proteins located in osteoarthritic synovial fluid was conducted and 40 proteins which were described to play a role in OA were identified. The 40 measured proteins include: IL-1β, IL-6Ra, IL-2, IL-5, IL-7, IL-12, IL-15, IL-17a, IL-18, IL-22, IL-23, IL-31, CCL-1, CCL-2/MCP-1, CCL-3/MIP-1α, CCL-5/RANTES, CXCL-8/IL-8, CXCL-10/IP-10, fractalkine/CX3CL1, CD-163, TNFα, OSM, LIF, resistin/ADSF, VEGF-A, IL-1Ra, IL-4Ra, IL-10, IL-13, MMP-1, MMP-2, MMP-3, MMP-9, MMP-13, COL1A1, ACAN, ON/SPARC, IFNγ, IL-33, TRANCE/RANKL. A custom Human Magnetic Luminex Assay (R&D System Inc., Minneapolis, MN, USA) was designed, and all 40 factors were quantified in the biological samples by Magpix Luminex Xmap Technology (Thermofisher Scientific, Waltham, MA, USA), previous treating the samples with hyaluronidase enzyme solution (Sigma, St. Louis, MO, USA, H4272-30 mg) in order to reduce the viscosity of HA. Differential protein level “before treatment” and “2 weeks after treatment” was calculated and correlated to the rest of proteins. Lastly, Multivariate Factor Analysis was performed with the software Statistica (version 9, StatSoft, Hamburg, Germany).

### 2.4. Statistical Analysis

Data were analyzed with one-way analysis of variance (ANOVA) and Tukey’s post hoc test, Pearson’s correlation and factor analysis. Prism 8 software (Irvine, CA, USA), SPSS was used for statistical analysis. Significance level was *p* > 0.05. Data are presented as mean + SEM.

## 3. Results

**Figure 3:**
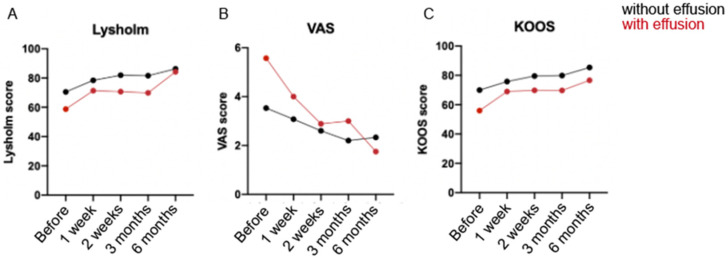
Graphs showing the Lysholm-Tegner (**A**), VAS (**B**) and KOOS (**C**) scores. Clinical data after 6-month follow-up after hyperacute serum treatment in OA knee at different time points: before the treatment, and after it at week 1, week 2, 3 months and 6 months. Red represents the clinical data from patients with effusion in the knees and black, without effusion.

To investigate whether hyperacute serum directly induced benefits in OA knees, patients were monitored up to 6 months and the effects were evaluated with patient reported outcomes, focusing on pain reduction and mobility improvement in everyday tasks and sports. It is known that occurrence of knee effusion is more common at an early stage of the disease; therefore, and due to the physiological differences between patients, they were divided into two subgroups: “patients without effusion” (also called “dry knee”) (*n* = 13) and “patients with effusion” (*n* = 9). For both groups, hyperacute serum demonstrated a steady improvement when examining patient’s knee instability, pain, swelling, mechanical locking, stair climbing and squatting, as shown with an increasing Lysholm-Tegner score over time (*p* < 0.001). The subgroup of “patients without effusion”, had lower pain level and better mobility by the beginning of the therapy when compared to patients with effused knee. Nevertheless, after 6 months of hyperacute serum treatment, pain and mobility were nearly the same for both subgroups. Same positive outcome was confirmed over time by Visual Analog Scale (VAS) and Knee Injury and Osteoarthritis Outcome Score (KOOS) measurements (*p* = 0.026 and *p* = 0.006, respectively) (Figure 3).

From the 24 patients investigated only 9 yielded before-after synovial fluid samples. This is partly due to the fact that in the early stages of OA the joint is not yet effused (‘dry painful knee’) or that some samples were stained with blood. Moreover, 6 months after treatment, sample size was 7, as 2 patients improved considerably, and it was not possible to obtain synovial fluid from them anymore. We investigated whether there were any significant changes in the cytokine levels of the patients before and after treatment. Although no individual differences in the mean cytokine level of the patients over time (considering the measurements before treatment, at week 1 and at week 2) could be identified, probably due to the low sample size (Suppl. Table S1); according to the multivariate general linear model Roy’s Largest Root, there was a significant change in the cytokine levels as a result of the serum therapy at week 2 (*p* = 0.023).

**Figure 4:**
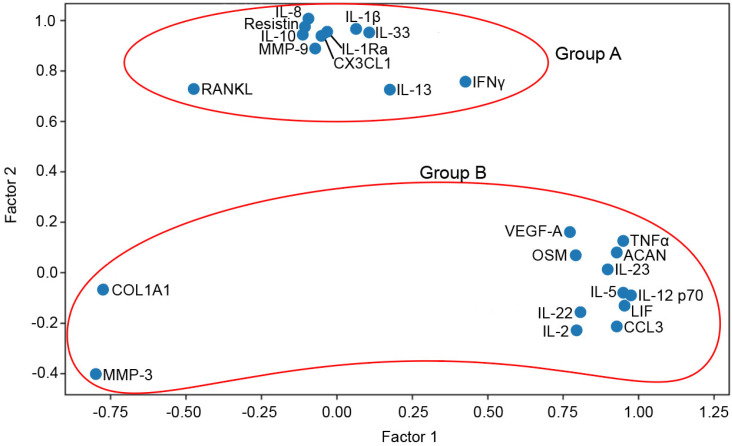
Subgroups of investigated cytokines (*n* = 24) after principal component analysis. The factor analysis suggested 2 main factors one containing 11 and other 13 cytokines. With the help of the factor analysis, we were able to reduce the number of variables.

Multivariate Factor Analysis was used to investigate clusters of cytokines that may react similarly to a common factor (Figure 4). Using the data from week 2 and based on Eigenvalue score, 2 main groups, which included 24 out of the total 40 measured cytokines, were identified. The 11 molecules clustered in Group A included the proinflammatory cytokines IL-1β, IL-13 and IL-33, the anti-inflammatory cytokine IL-10; the IL-1 receptor antagonist, IL-1Ra; chemokines such as IL-8 and fractalkine (CX3CL1); the matrix metalloproteinase MMP-9; the peptide hormone resistin; the cytokine IFNγ and the ligand for the RANK receptor (RANKL), involved in bone resorption through osteoclast activation. According to the Multivariate Factor Analysis performed, Group B was more disperse and therefore less homogeneous in terms of similar reaction patterns to hyperacute serum. Group B included the proinflammatory cytokines TNFα, IL-5, IL-2, IL-12p70, IL-22 and IL-23; metalloproteinase MMP-3; chemokine CCL3(MIP1α); the pleotropic cytokines leukemia inhibitory factor (LIF) and oncostatin (OSM); and other proteins that play a role in inflammatory processes and extracellular matrix structural support, such as the vascular endothelial growth factor A (VEGF-A), aggrecan (ACAN) and collagen type I α (COL1A1).

**Figure 5:**
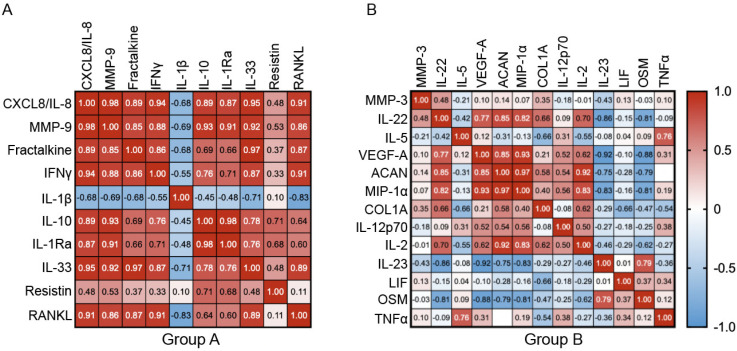
Pearson’s correlation heatmaps of the two identified protein groups after Multivariate Factor Analysis. Cytokines belonging to group A (left panel), presented strong to very strong correlation (*p* < 0.05), with the exception of resistin (**A**). Generally, group B (right panel) presented weaker correlations; however, the cluster including VEGF-A, ACAN, CCL3/MIP-1α, IL-22, IL-2, IL-23 and OSM were highly and significantly correlated (*p* < 0.05) (**B**). Color code represents positive or negative linear relationship between proteins, red and blue, respectively. White values indicate no shared trend between the proteins.

To further understand a possible correlation between the cytokines identified within the two groups, Pearson correlation Heatmap of hyperacute serum-induced changes was calculated (Figure 5). Group A showed a strong and significant correlated pattern between all cytokines, except for resistin (most r^2^ > 0.75, as represented in Figure 5A). Strikingly, only IL-1β correlated negatively. On the other hand, in group B most of the correlations were weak as shown in Figure 5B. However, a subgroup consisting of VEGF-A, ACAN, MIP-1α, IL-22, IL-2, IL-23 and OSM presented a strong correlation with each other (most r^2^ > 0.85), suggesting that not all group B, but these seven proteins behave similarly after hyperacute serum IA treatment (Figure 5B).

**Figure 6:**
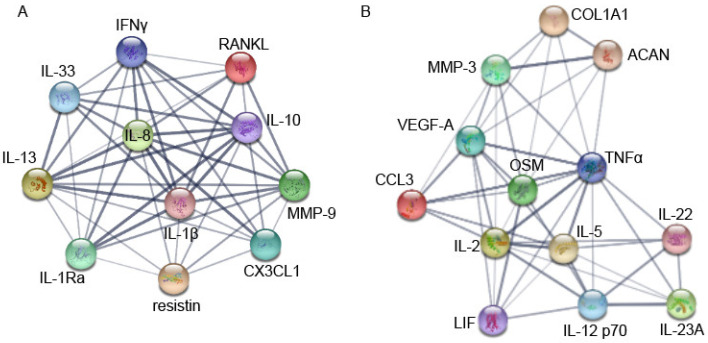
Protein–protein interaction analysis of the two identified groups by STRING database. Nodes represent proteins and the lines represent known interactions between the linked two proteins. The thickness of the lines indicates the confidence of the data which supports this interaction. Groups (**A**,**B**) are represented in the left and right panels, respectively. Color of nodes does not give information.

This high correlation suggests that cytokines belonging to the same group or cluster may fluctuate together. To gain a better understanding in known protein–protein interactions of the two groups, further network analysis using the STRING database was performed (Figure 6). STRING analysis showed that connectivity rates were slightly higher for group A (Figure 6A) as it had 47 connections between 11 proteins; and group B (Figure 6B) revealed 47 connections between 13 proteins, resulting in 4.27 and 3.61 of connectivity rate, respectively. Moreover, when looking at the confidence of the data supporting those connections, represented by the thickness of the lines, group A was higher when compared to group B.

## 4. Discussion

This is the first pilot study of IA hyperacute serum interventions in OA patients. In this study, safety and feasibility of the intervention was verified showing that during the 6 months of follow up clinical meetings, no adverse event that would cause any expected or unexpected risk were reported. Minor observations produced by the invasiveness of the procedure have been described for such IA infiltrations of PRP and HA; however, the possible mild local pain spontaneously disappears within 2 days [2,65]. When looking at other blood-derived products, clinical trials have shown that PRP provides a significant improvement in pain relief after knee or lumbar intradiscal injections [66,67]. Same positive trend was observed while comparing PRP to HA [68]; however, different outcomes depending on the trial setup arise controversy in this comparison. Di Martino et al. showed that the positive effect of PRP in OA knees, was only significantly better than HA after 24 months of follow-up, and it failed to be significant after longer periods of time [69]. As previously mentioned, the different PRP preparation techniques result in a high variability of the product, leading to an unpredictable and inconclusive outcome [21]. Hyperacute serum overcomes this handicap and adds a different composition in growth factors and cytokines with high potential in in vitro tissue regeneration [19,25], becoming a promising therapeutic in OA.

Synovial fluid is easily harvested from synovial joints and commonly used in research for marker identification in OA [70]. As shown elsewhere, protein patterns of the synovial fluid allow for the identification of distinct pathologies, which may allow patient selection and targeted therapies in the future, focusing on the therapeutic use of biologics to patients who can truly benefit from the treatment [71,72]. In this study, synovial fluid from OA patients was collected prior the first IA hyperacute serum injection and after 1 and 2 weeks of the administration. At week 2 after the first treatment, 2 out of the 9 patients from whom synovial fluid was collected, improved significantly and the extraction of excessed synovial fluid was not possible. Forty known OA-related proteins were analyzed over time by performing a Multivariate Factor Analysis showing that 24 of them clustered into 2 defined groups, indicating that these proteins may influence each other. Further statistical tests showed a high correlation within those groups suggesting that the response of some of those cytokines could be predictable. This interesting finding may allow us to reduce the number of the investigated cytokines in future studies, extrapolating to the rest of the group and, therefore, not losing important information. It could be used to follow up OA patients’ response to treatments, also during clinical trials, optimizing biological samples and experimental resources. This innovative approach was already described by *Zhang* et al. and performed for other diseases such as breast cancer [73,74].

Group A, except for resistin, revealed an outstandingly strong and significant correlation when comparing fluctuations during hyperacute serum treatment. Previous correlations between these cytokines have been already reported. For example, the activation pattern of IFNγ, IL-10 and IL-33, playing a role in osteoclast downregulation, had been previously linked [75,76]. Furthermore, IL-8 increases the level of MMP-9 in the context of the autoimmune disease Epidermolysis Bullosa Simplex [77]. Interestingly, the only cytokine in this group which correlated negatively was IL-1β, which is a well-known cytokine in OA. Although, being able to induce cartilage degradation through the activation of metalloproteinases, it has been shown in multiple clinical trials that it is not an optimal target in the treatment of OA [78,79,80,81]. On the other hand, for Group B most of the correlations were too weak and irrelevant. It is of importance to highlight the seven proteins where the correlation was strong and significant: VEGF-A, ACAN, MIP-1α, IL-22, IL-2, IL-23 and OSM; these last two correlating negatively. It is known that VEGF-A plays a crucial role in the onset of OA, resulting in cartilage degeneration due to inhibition of synthesis of ACAN [82,83]. Moreover, although correlation in OA between MIP-1α, IL-22 and IL-2 has not been previously described, it is known that they induce inflammation; and the presence of citrullinated ACAN in rheumatoid arthritis results in the upregulation of IL-2 and IL-22 [84]. Similarly to our study, *Botta* et al. developed an inflammatory profile for multiple myeloma where the downregulation of MIP-1α and VEGF-A predicted a better prognosis of the disease [85]. Interestingly, it is known that both OSM and IL-23 are upregulated in OA joints, having a direct effect in the inhibition of GAG production and in pain, respectively [86,87]; however, as far as our knowledge goes, there is no proven correlation between these two proteins despite behaving similarly after hyperacute serum treatment.

As a pilot study, and due to the lack of a control group, the efficacy of the treatment could not be proven. Nevertheless, patient reported outcomes were gathered up to 6 months of follow-up showing that the quality of life of treated patients improved over time significantly. During the first stages of OA, it is common that joints present an excess of synovial fluid, also called joints “with effusion” [88]. We observed that patients which such condition were more compromised. Nevertheless, the two groups of OA patients (“with” and “without effusion”) had a positive tendency and the patients reported outcomes were promising both on short and long-term effects. With the help of hyperacute serum therapy, pain level was reduced, and mobility improved. However, as an open-label study, it is not possible to ensure that hyperacute serum triggers a significantly better outcome than the placebo effect. It is well known that placebo response in OA has a considerable effect and has been widely documented, showing a significant and sometimes, a greater effect, especially in pain alleviation, when compared to different treatments in randomized trials [89,90]. Moreover, it has been shown that for IA interventions, placebo effect is even more notable when compared with less invasive and less frequent medical procedures [8,91,92]. Taking into account these considerations and the promising results observed; there is a need for a randomized controlled clinical trial of hyperacute serum to determine best candidates for this treatment, administration intervals and hyperacute serum efficacy. For this reason, it would be of interest including a comparison to certain established treatments, such as PRP, to evaluate a possible superior efficacy, as suggested by experimental data.

## 5. Conclusions

We conclude that, after hyperacute serum treatment, no adverse events were reported, proving the safety of the therapy. Hyperacute serum treatment showed a positive effect in alleviating symptoms and providing an improvement in osteoarthritic knees in the context of pain and functionality, probably by inducing regenerative processes, especially for the patients with “effusion knees”. In this study, although no significant individual protein changes were identified from synovial fluid samples, two groups of proteins showing highly correlated fluctuations were detected after hyperacute serum treatment, suggesting that those two clusters behave as two groups; this fact was supported by protein–protein interaction analysis. Therefore, the possibility of measuring the levels some of the group members to estimate the behavior of the whole group opens new possibilities during treatment follow-up.

## Supplementary Materials

The following are available online at https://www.mdpi.com/article/10.3390/cimb43020046/s1. Table S1: List of the 40 measured cytokines and OA-related proteins with the mean expressed levels and standard errors. Table S2: Inclusion and exclusion criteria of osteoarthritic patients included in this hyperacute serum IA injection trial. Table S3. Patient information.
