# Supplementary material for: A Pilot Clinical Study of Hyperacute Serum Treatment in Osteoarthritic Knee Joint: Cytokine Changes and Clinical Effects
Source: Curr Issues Mol Biol. 2021 Jul 9;43(2):637–49. doi: 10.3390/cimb43020046 (PMC8929160; doi:10.3390/cimb43020046)
Supplement: Supplementary file 1 [file cimb-43-00046-s001.zip › cimb-1268318-supplementary.pdf]

Article Title: A Pilot Clinical Study of Hyperacute Serum Treatment in Osteoarthritic Knee Joint: Cytokine Changes and Clinical Effects

Journal name: Knee Surgery, Sports Traumatology, Arthroscopy

Name of the Authors: Isabel Olmos Calvo 1,*, Eszter Fodor 2, Dorottya Kardos 3, István Hornyák 3, Adél Hinsenkamp 3, Olga Kuten-Pella 1, Zsuzsanna Gyevnár 2, Gábor Erdélyi 4, Tamás Bárdos 4, Tamás Mirkó Paukovits 4, Krisztián Magos 4, György Béres 4, Stefan Nehrer 5 and Zsombor Lacza 2,3

Affiliations:

[1]OrthoSera GmbH, Dr. Karl-Dorrek-Straße 23-29, 3500 Krems an der Donau, Austria.
[2]Department of Sport Physiology, University of Physical Education, 44 Alkotás utca, 1123 Budapest, Hungary.
[3]Institute of Translational Medicine, Semmelweis University, 26 Üllöi utca, 1085 Budapest, Hungary.
[4]Kastélypark Klinika, 15 Hajdú utca, 2890 Tata, Hungary.
[5]Center for Regenerative Medicine, Danube University, Dr. Karl-Dorrek-Straße 30, 3500 Krems an der Donau, Austria.

*Correspondence: isabel.olmos@orthosera.com

SUPPLEMENTARY DATA

Table S1: List of the 39 measured cytokines and OA-related proteins with the mean expressed levels and standard errors (SEM).

Abbreviation	Name		Concentration (pg/ml)	SEM	Description
		**1. Proinflammatory interleukins**			
**IL-1β**	**Interleukin-1 β**	week 0	8,830	0,922	Proinflammatoty cytokine; positive correlation with severity and with joint space; biomarker of the burden of OA; biomarker of the efficacy of intervention
		week 1	8,566	1,179	
		week 2	8,828	1,030	
**IL-6 Ra**	**Interleukin-6 receptor antagonist**	week 0	17 476,355	1 543,565	Healthy chondrocytes produce low amount of IL-6; it inhibits type II collagen synthesis; plays a role in cartilage degradation; associates with an increased prevalence of osteophytes; it
		week 1	16 370,347	1 186,035	
		week 2	15726,555	1271,857	

					causes changes in subchondral bone layer; diagnostic and prognostic biomarker of OA
**IL-2**	**Interleukin-2**	week 0<br>week 1<br>week 2	179,208<br>204,048<br>178,544	12,818<br>35,778<br>13,858	Significant concentration differences in IL-2, IL-5, MCP-1, and MIP-1 were found between subjects with advanced arthritis and subjects with little or no arthritis on the ICRS scale; IL-2 was only detected in four of the end-stage synovial fluid specimens
**IL-5**	**Interleukin-5**	week 0<br>week 1<br>week 2	19,945<br>17,074<br>16,703	4,422<br>2,713<br>2,763	Produced by Th2 cells and mast cells; higher concentration in advanced OA; correlates with ICRS scale
**IL-7**	**Interleukin-7**	week 0<br>week 1<br>week 2	26,235<br>25,930<br>26,335	1,281<br>0,983<br>1,097	Chondrocytes express IL-7 receptor and increase MMP and proteoglycan production after IL-7 stimulation; it increases with age in synovial fluid samples from OA patients; diagnostic biomarker of OA
**IL-12**	**Interleukin-12**	week 0<br>week 1<br>week 2	180,955<br>184,952<br>183,987	23,428<br>25,240<br>25,846	Proinflammatory cytokine expressed by tissue infiltrated leukocytes; it shows an increased level in OA
**IL-15**	**Interleukin-15**	week 0<br>week 1<br>week 2	19,166<br>20,373<br>18,708	1,677<br>1,913<br>2,241	Proinflammatory cytokine which contributes to inflammation in OA; it has an independent positive correlation

					with WOMAC pain scores; potential diagnostic of OA, biomarker of OA in early disease development and burden.
**IL-17a**	**Interleukin-17a**	week 0	27,478	2,206	Inflammatory cytokine which affects chondrocytes by increasing the production of MMPs and inhibiting proteoglycan synthesis; positive correlation with radiographic images of lesions in OA
		week 1	31,571	2,909	
		week 2	30,156	2,162	
**IL-18**	**Interleukin-18**	week 0	117,877	11,395	Positive correlation with disease severity; diagnostic biomarker of OA
		week 1	121,468	12,585	
		week 2	125,950	12,430	
**IL-22**	**Interleukin-22**	week 0	202,696	34,301	Proinflammatory cytokine secreted by Th17, Th1, NK, NKT cells. Inflamed areas were associated with higher expression of IL-17 and IL-22, both correlated with the combined release of IL-6, IL-23, and TGFβ1; responsible of inflammation at the synovium
		week 1	170,649	23,132	
		week 2	93,897	36,373	
**IL-23**	**Interleukin-23**	week 0	1 487,900	161,345	Proinflammatory cytokine expressed in subchondral bone marrow and in fibrous tissue replacing the marrow, potential therapeutic target in OA
		week 1	1 536,334	134,462	
		week 2	1313,739	98,164	
**IL-31**	**Interleukin-31**	week 0	24,409	1,032	Released by monocytes, macrophages, T
		week 1	24,156	1,018	

		week 2	23,462	0,817	cells and dentritic cells, IL-31 may function as a proinflammatory cytokine involved in the recruitment of polymorphonuclear cells
**2. Proinflammatory chemokines**					
**CCL-1**	**Chemokine (C-C motif) ligand 1**	week 0	3,223	0,368	High plasma concentration found in OA: it increases the expression of MMP-3 and MMP-13
		week 1	3,140	0,286	
		week 2	2,869	0,235	
**CCL-2/MCP-1**	**Chemokine (C-C motif) ligand 2 / Monocyte chemotactic protein 1**	week 0	867,635	115,981	It induces MMP3 expression and inhibits proteoglycan synthesis
		week 1	787,776	78,802	
		week 2	918,578	165,321	
**CCL-3/MIP-1α OA**	**Chemokine (C-C motif) ligand 3 / Macrophage inflammatory protein 1- alpha**	week 0	261,050	13,872	Potential serum OA biomarker; it stages the severity of joint damage
		week 1	268,700	11,794	
		week 2	267,485	18,062	
**CCL-5/RANTES**	**Chemokine (C-C motif) ligand 5 /Regulated upon activation, normal T cell expressed and secreted**	week 0	110,326	23,982	High concentration in OA synovial fluid; positive correlation with MMP-2, IL-6, IL-8 and MMP-1
		week 1	98,407	18,770	
		week 2	85,270	10,279	
**CXCL-8/IL-8**	**chemokine (C-X-C motif) ligand 8/ Interleukin-8**	week 0	619,984	459,445	Stimulates osteoclastogenesis and bone resorption; depresses the activity of osteoblasts; significantly increased levels in synovial fluid of OA patients; it can explain bone loss and cartilage injury in OA
		week 1	228,681	131,866	
		week 2	178,606	74,621	
**CXCL-10/IP-10**	**Chemokine (C-X-C motif) ligand-10/Interferon gamma-induced protein 10**	week 0	254,469	98,628	May play a role in the pathophysiology of OA; levels found in plasma and synovial fluid
		week 1	270,975	69,819	
		week 2	171,303	27,743	

					correlates with radiographic severity in knee OA
**Fractalkine/ CX3CL1**	**Fractalkine/chemokine (C-X3-C motif) ligand 1**	week 0	1 351,828	483,418	Fractalkine in synovial fluid and serum may serve as a OA biomarker for reflecting symptomatic severity; it regulates OA fibroblast migration
		week 1	254,469	98,628	
		week 2	1302,739	469,306	
**3. Other inflammatory proteins**					
**CD-163**	**Cluster of Differentiation 163**	week 0	1 876 059,862	118 418,722	Soluble macrophage biomarker that reflects the abundance of activated macrophages; it correlates with structural progression, and together with CD14, pain of OA
		week 1	1 791 750,028	128 284,187	
		week 2	1776789,080	120059,479	
**TNFα**	**Tumor necrosis factor alpha**	week 0	12,441	0,896	It increases the synthesis of IL-6, IL-8, RANTES and VEGF; it shows a positive correlation with pain; OA burden of disease biomarker and biomarker of efficacy of intervention
		week 1	12,414	0,742	
		week 2	11,819	0,819	
**OSM**	**Oncostatin-M**	week 0	1 293,207	154,720	Contributes to the pathogenesis of OA as overexpression of OSM induces joint damage due to cartilage destruction; it induces osteophyte formation
		week 1	1 113,583	93,311	
		week 2	1088,947	73,334	
**LIF**	**Leukemia inhibitor factor**	week 0	41,744	3,009	It degrades proteoglycans and regulates bone formation and resorption; Biomarker of OA
		week 1	42,657	2,748	
		week 2	41,827	2,956	

					(together with IL-11 and OP-1, while IL-1, IL-6, IL-8 are biomarkers of RA)
**Resistin/ ADSF**	**Resistin/adipose tissue-specific secretory factor**	week 0<br>week 1<br>week 2	7 634,535<br>11 547,598<br>7090,042	4 392,447<br>8 823,966<br>4927,750	Resistin is present in OA joints and is released from OA cartilage. Levels of resistin in synovial fluid are associated with inflammatory and catabolic factors and may serve as a potential biomarker for reflecting the disease severity and cartilage degenerative extent of knee OA
**VEFG-A**	**Vascular endothelial growth factor A**	week 0<br>week 1<br>week 2	658,332<br>565,015<br>685,542	117,431<br>66,860<br>131,738	Produced by hypotrophic chondrocytes, macrophages and synovial fibroblasts. Levels in synovial fluid and plasma correlates with OA severity
**4. Anti-inflammatory interleukins**					
**IL-1 Ra**	**Interleukin-1 receptor antagonist**	week 0<br>week 1<br>week 2	10 197,060<br>1 280,844<br>1001,694	9 044,266<br>623,155<br>284,733	It uses the receptor of IL-1, a proinflammatory cytokines, which has pivotal role in the pathogenesis of OA. IL-1Ra opens a promising therapeutic perspective for patients with OA
**IL-4 Ra**	**Interleukin-4 receptor antagonist**	week 0<br>week 1<br>week2	453,143<br>457,529<br>453,1089644	29,728<br>27,680<br>35,9126296	It uses the receptor of IL-4, an anti-inflammatory cytokine, which alone or together with IL-10, inhibits the apoptosis of chondrocytes; IL-4Ra shows elevated

					levels in OA serum and decreases the synthesis of IL-1β, TNFα, IL-6, PGE2 and iNOS
**IL-10**	**Interleukin-10**	week 0<br>week 1<br>week 2	4,892<br>4,824<br>5,027	0,513<br>0,503<br>0,464	Anti-inflammatory cytokine that, together with IL-4, inhibits the apoptosis of chondrocytes; it stimulates the synthesis of collagen type II and ACAN, inhibits MMPs; it may serve as a prognostic biomarker
**IL-13**	**Interleukin-13**	week 0<br>week 1<br>week 2	990,858<br>935,396<br>981,725	112,237<br>107,221<br>112,207	It is a potentially useful marker in the therapeutic treatment of OA as it can reduce the production of proinflammatory cytokines, MMPs and induce the production of IL-1Ra
**5. Matrix metallorpoteases (MMPs)**					
**MMP-1**	**Matrix metalloproteinase-1**	week 0<br>week 1<br>week 2	20 864,725<br>17 428,380<br>21135,543	3 445,281<br>3 139,476<br>3758,370	Higher concentration in OA synovial fluid than in healthy donor's; it is mainly found in superficial cartilage promoting cartilage degradation; it shows positive correlation with MMP-2, IL-6, IL-8, CCL-5 and radiographic scores
**MMP-2**	**Matrix metalloproteinase-2**	week 0<br>week 1<br>week 2	54 494,074<br>56 489,352<br>56088,676	873,746<br>984,161<br>1289,565	Higher concentration in OA synovial fluid than in healthy donor's inducing cartilage degradation; it shows positive

					correlation with MMP-1
**MMP-3**	**Matrix metalloproteinase-3**	week 0	44 835,023	3 017,750	Higher concentration in OA synovial fluid than in healthy donor's inducing cartilage degradation; it shows positive correlation with OA severity
		week 1	43 226,792	2 951,829	
		week 2	42943,606	2390,735	
**MMP-9**	**Matrix metalloproteinase-9**	week 0	5693,470	3405,017	Higher concentration in OA synovial fluid than in healthy donor's inducing cartilage degradation
		week 1	7 892,046	6 695,381	
		week 2	2992,058	2033,618	
**MMP-13**	**Matrix metalloproteinase-13**	week 0	1 093,847	1 106,511	It degrades cartilage, mainly type II and IX collagen; it is found in higher concentration in deep layers of OA cartilage; overexpressed in early OA and down-regulated in late stage OA; it correlates with OA lesion severity
		week 1	27,382	21,486	
		week 2	1122,871	29,899	
**6. Cartilage and bone remodeling markers**					
**COL1A1**	**Collagen type I α 1 chain**	week 0	28 685,383	1 837,766	COL1A1 defect leads to rapidly progressive OA in mouse models
		week 1	27 665,064	1 885,586	
		week 2	28042,61107	1625,810876	
**ACAN**	**Aggrecan**	week 0	923,693	108,034	Suitable for the monitoring of cartilage damage; it shows positive correlation with joint damage but not with clinical parameters
		week 1	936,220	110,573	
		week 2	940,339	128,716	
**ON/SPARC**		week 0	301 735,075	38 193,107	TGF β 1 and IL-1 β, regulate the production of
		week 1	317 977,738	61 439,522	

	**Osteonectin/secreted protein acidic and rich in cysteine**	week 2	311872,596	82818,26005	SPARC by chondrocytes at pre- and posttranslational levels; SPARC synthesis is markedly enhanced in arthritic joints and it is associated with radiographic OA status (except previous meniscectomy) when found in synovial fluid
**7. Osteoarthritis biomarkers, which are extremely high in Rheumatoid Arthritis**					
**IFNγ**	**Interferon gamma**	week 0	38,892	12,920	It inhibits IL-1-induced MMP-3 expression in healthy chondrocytes; decreased IFNγ level in OA chondrocytes in contrast to higher concentration in RA
		week 1	34,541	14,151	
		week 2	27,071	4,315	
**IL-33**	**Interleukin-33**	week 0	24,409	7,827	Inflammatory cytokine; potential marker of RA
		week 1	23,111	8,986	
		week 2	15,710	2,288	
**TRANCE/RANK L**	**TNF-related activation-induced cytokine/Receptor activator of nuclear factor kappa-B ligand**	week 0	98,463	34,520	OPG/RANKL/RANK system is important in the balance between bone formation and resorption; it plays a role in subchondral bone metabolism; it is involved in OA progression
		week 1	96,707	39,183	
		week 2	61,864	9,606	

Table S2. Inclusion and exclusion criteria of osteoarthritic patients included in this hyperacute serum IA injection trial.

Inclusion Criteria	Exclusion Criteria
1. Presence of early or low-grade knee OA with KL = 2 or 3 in the index knee evidenced by standing radiographs and clinical examination up to 3 months before the study treatment.	1. Body Mass Index >40
2. Male or female ≥18 and ≤70 years old at time of injection.	2. Any documented clinically significant condition (e.g., diabetes, malignancy), finding, or psychiatric illness at screening which could compromise patient safety or interfere with the assessment of the safety and treatment effects of the study injection.
3. Minimum 4 points from the maximum 10 points on VAS scale up to 3 months before the study treatment, and the pain is caused by OA.	3. Any intra-articular therapy in the index knee within 6 months prior to screening, systemic steroid use within 3 weeks of screening.
4. Clinical examination, which confirms, that the etiology of the pain is attributable to OA	4. Diagnosed with rheumatoid arthritis, Reiter's syndrome, psoriatic arthritis, gout, ankylosing spondylitis, chondromalacia, arthritis secondary to other inflammatory diseases, or of metabolic origin; HIV, viral hepatitis; chondrocalcinosis, Paget's disease, villonodular synovitis, and other non-OA joint disease.
5. Inflammatory arthropathy is not presented.	5. Presence of active infection or massive acute inflammation in the knee immediately preceding treatment injection.
6. In case of chronic liver or kidney diseases certificate is needed about the stabile state of the disease during the last 6 months.	6. Moderate and severe anemia, thrombocytopenia or polycythemia (Hemoglobin >17 g/dl or <11 g/dl and/or platelet number >500,000 or <100,000 /μl.)
	7. Patients with untreated renal or kidney dysfunction and dialysis patients.
	8. Pregnant or nursing mothers or women who are planning on getting pregnant during the time they will be participating in the study.
	9. Known drug or alcohol dependence currently or within the last year.
	10. Patients under therapeutic doses of anticoagulant therapy (e.g. Warfarin, Dabigatran, Enoxaparin) and patients with coagulopathy. Patients with platelet aggregation inhibitory therapy (e.g. aspirin, clopidogrel) are not excluded.
	11. Lack of opportunity to travel (e.g. there and back to checkups) or lack of co-operation to participate in treatments / examinations.

	12. An advanced or acute phase malignancy.
	13. Blood disorders (e.g. sickle cell anemia).

Table S3. Patient information.

Number of patients (n)	24
Female sex (n)	13
Mean age (years), standard deviation (years)	49,67 ± 9,54
